# The changing epidemiology of bacillary dysentery and characteristics of antimicrobial resistance of *Shigella *isolated in China from 2004–2014

**DOI:** 10.1186/s12879-016-1977-1

**Published:** 2016-11-18

**Authors:** Zhaorui Chang, Jing Zhang, Lu Ran, Junling Sun, Fengfeng Liu, Li Luo, Lingjia Zeng, Liping Wang, Zhongjie Li, Hongjie Yu, Qiaohong Liao

**Affiliations:** Division of Infectious Diseases, Key Laboratory of Surveillance and Early-warning on Infectious Disease, Chinese Center for Disease Control and Prevention, 155 Changbai Road, Changping District, Beijing, 102206 People’s Republic of China

**Keywords:** Epidemiology, Bacillary dysentery, Resistance, *Shigella*

## Abstract

**Background:**

Bacillary dysentery caused by bacteria of the genus *Shigella* is a significant public health problem in developing countries such as China. The objective of this study was to analyze the epidemiological pattern of bacillary dysentery, the diversity of the causative agent, and the antimicrobial resistance patterns of *Shigella* spp. for the purpose of determining the most effective allocation of resources and prioritization of interventions.

**Methods:**

Surveillance data were acquired from the National Infectious Disease Information Reporting System (2004–2014) and from the sentinel hospital-based surveillance system (2005–2014). We analyzed the spatial and temporal distribution of bacillary dysentery, age and sex distribution, species diversity, and antimicrobial resistance patterns of *Shigella* spp.

**Results:**

The surveillance registry included over 3 million probable cases of bacillary dysentery during the period 2004–2014. The annual incidence rate of bacillary dysentery decreased from 38.03 cases per 100,000 person-years in 2004 to 11.24 cases per 100,000 person-years in 2014. The case-fatality rate decreased from 0.028% in 2004 to 0.003% in 2014. Children aged <1 year and 1–4 years were most affected, with higher incidence rates (228.59 cases per 100,000 person-years and 92.58 cases per 100,000 person-years respectively). The annual epidemic season occurred between June and September. A higher incidence rate of bacillary dysentery was found in the Northwest region, Beijing and Tianjin during the study period. *Shigella flexneri* was the most prevalent species that caused bacillary dysentery in China (63.86%), followed by *Shigella sonnei* (34.89%). *Shigella* isolates were highly resistant to nalidixic acid (89.13%), ampicillin (88.90%), tetracycline (88.43%), and sulfamethoxazole (82.92%). During the study period, isolates resistant to ciprofloxacin and cefotaxime increased from 8.53 and 7.87% in 2005 to 44.65 and 29.94% in 2014, respectively.

**Conclusions:**

The incidence rate of bacillary dysentery has undergone an obvious decrease from 2004 to 2014. Priority interventions should be delivered to populations in northwest China and to individuals aged <5 years. Antimicrobial resistance of *Shigella* is a serious public health problem and it is important to consider the susceptibility profile of isolates before determining treatment.

## Background

Bacillary dysentery, which is primarily transmitted by the fecal-oral route via contaminated food, water, or person-to-person contact [1–5], is an important enteric infectious disease caused by *Shigella* spp. The major symptoms of bacillary dysentery include diarrhea, fever, abdominal pains, tenesmus and stool with blood or mucus [6]. Annually, there are 165 million reported or confirmed cases of bacillary dysentery and 1.1 million deaths worldwide, predominantly in developing countries [7]. In mainland China, the annual morbidity and mortality of bacillary dysentery ranked in the top ten of 39 notifiable infectious diseases from 2004 to 2014.

Bacillary dysentery can be caused by four *Shigella *species: *S. dysenteriae, S. flexneri, S. boydii,* and *S. sonnei* [8]*. Shigella sonnei* is the most prevalent *Shigella* species in developed countries. In developing countries, *S. flexneri* tends to be predominant, while *S. dysenteriae* and *S. boydii* are comparatively rare [7]*.* With the overuse of antibiotics, antimicrobial resistance has been increasing among *Shigella* isolates in recent years, limiting the possibilities for suitable empirical antibiotic treatments [9]. Knowledge of the disease burden and epidemiological characteristics of bacillary dysentery is helpful for allocating resources and prioritizing interventions. Therefore, in this study, we investigated the temporal and spatial distribution of bacillary dysentery, determined the high-risk populations of bacillary dysentery, and determined the predominant circulating species and antimicrobial resistance patterns of *Shigella* based on existing data sources from the National Infectious Disease Information Reporting System (NIDRS) from 2004 to 2014 and the sentinel-based bacillary dysentery surveillance system from 2005 to 2014 in China.

## Methods

### National surveillance for bacillary dysentery

Bacillary dysentery was added to the list of notifiable diseases in China in 1956. From 1956 to 2003, the number of cases and deaths by province were reported monthly to the Chinese Center for Disease Control and Prevention (China CDC). After 2004, the NIDRS, an internet-based notifiable infectious disease reporting system, was established [10]. Thereafter, all probable and confirmed cases along with individual data were reported online to the China CDC by clinicians within 24 h of diagnosis. Clinicians diagnose bacillary dysentery cases according to the unified diagnosis criteria issued by the Chinese Ministry of Health. A probable case of bacillary dysentery was defined as a patient with the following clinical features: fever, chills, abdominal pain, tenesmus, bloody or mucus stool or stool containing >15/high power field (HPF) leukocytes or purulent cells, and microscopically discernible red blood cells and phagocytic cells. A confirmed case was defined as a patient with *Shigella* spp*.* isolated from a stool specimen [11]. The individual data includes gender, date of birth, address, case classification (probable or confirmed), date of onset, and date of death (if applicable). All data used in this study for bacillary dysentery cases reported from 1 January 2004 to 31 December 2014 in China were acquired from the NIDRS.

### Sentinel hospital-based bacillary dysentery surveillance

Sentinel hospital-based bacillary dysentery surveillance was established in 2005 to monitor the predominant circulating species and antimicrobial resistance patterns of *Shigella* in China. The surveillance system consists of 20 sentinel hospitals distributed in Beijing, Gansu, Qinghai, Shanxi, Henan, Heilongjiang, Anhui, Fujian, Guizhou, and Shanghai. This coverage represents variation in geographical features, economic development, and sanitary conditions. A national surveillance protocol and laboratory testing assays were developed by the China CDC and are used by all sentinel sites [12].

### Specimen collection and testing

In each sentinel hospital, fecal specimens were collected from patients with diarrhea and clinically suspected dysentery who had not been given daily antimicrobial treatment. Fresh fecal samples were inoculated in Cary-Blair medium (most frequently, Qingdao Hope Bio-Technology Co., Ltd, Shandong, China) and sent to the regional CDC laboratory within 4 h. Stool samples were streaked onto XLD agar and SS agar and then incubated at 37 °C for 18–24 h. Colorless and transparent colonies were screened using triple sugar iron agar and motility indole-urea agar. Presumptive positive colonies were then confirmed using API 20E strips (bioMérieux, Marcy l’Etoile, France). All confirmed isolates were serotyped using commercial antisera (Denka Seiken Co. Ltd., Tokyo, Japan).

More than 300 fecal specimens were collected at each sentinel hospital every year, among which at least 30 specimens were selected for isolation and identification of bacteria each month in the epidemic season (May–October) and 10 specimens in the non-epidemic season (November–April). Sampling was designed to ensure that >50% of samples selected for isolation and identification of bacteria were from children.

### Antimicrobial susceptibility

The ability of different *Shigella* isolates to resist the inhibitory effects of different antibiotics was tested by the provincial CDCs. At least 30% of the confirmed *Shigella* isolates, which cover different species, were selected for antimicrobial susceptibility testing each month. Minimal inhibitory concentrations of the following nine antimicrobial agents were determined using the agar dilution method according to the Clinical and Laboratory Standards Institute (CLSI) guidelines [13]. As such, ampicillin, amoxicillin, cefotaxime, cephalothin, gentamicin, nalidixic acid, ciprofloxacin, tetracycline, sulfamethoxazole. *Escherichia coli* (American Type Culture Collection strain 25922) was used for quality control. Susceptible and non-susceptible isolates were identified according to the criteria used for enterobacteria as suggested by the CLSI.

### Data analysis

We included cases with illness onset reported to the NIDRS from 1 January 2004 to 31 December 2014 in the analysis. We not only calculated a crude incidence rate (number of cases divided by the corresponding population), a case-fatality rate (number of deaths divided by the number of probable and confirmed cases), and age-specific rates by sex (number of cases occurring in a specific age group divided by the corresponding population for each sex), but we also determined the seasonal patterns and geographic distribution of bacillary dysentery. Population data for the study period were extracted from the National Bureau of Statistics of China [14]. In addition, we analyzed the proportions of *Shigella* spp. isolated from laboratory-confirmed cases of bacillary dysentery and the resistance rates of *Shigella* collected from sentinel hospitals during 2005–2014. The Cochran-Armitage trend test was used to examine the temporal trends in the annual morbidity of bacillary dysentery, the proportion of bacillary dysentery cases by age groups, and the antimicrobial resistance of *Shigella* isolates. The index *Z* > 0 denotes an increasing trend in the annual morbidity of bacillary dysentery, the proportion of bacillary dysentery cases by age groups, and the antimicrobial resistance of *Shigella* isolates, whereas *Z* < 0 denotes a decreasing trend. The trend was considered to be significant when P was < 0.05. A chi-square test was used to examine whether the sex-specific incidence in children younger than 5 years was significantly different between male and female individuals, with a significance level of α = 0.05. We conducted all analyses with *SAS* 9.4 (SAS Institute Inc., Cary, USA).

We used a seasonal index to understand seasonal patterns of bacillary dysentery in China. This index was calculated by month as the average case count for the given month divided by the average monthly case count during the entire 11 years of surveillance from 2004 to 2014 [15]. No obvious seasonal pattern was expected if the seasonal index of each month was close to 1.0 [15].

The incidence rate of three time periods by province was used to demonstrate the geographic distribution of bacillary dysentery. The three time periods (2004–2006, 2007–2009, 2010–2014) were categorized according to the average incidence of bacillary dysentery (>30 per 100,000 person-years, 20–30 per 100,000 person-years and <20 per 100,000 person-years) in China. The software *ArcGIS* version 10.0 (ESRI, Redlands, CA, USA) was used to describe the spatial distribution of bacillary dysentery using a county-level polygon map.

## Results

### Temporal trend in incidence rate and case-fatality rate of bacillary dysentery

**Fig. 1:**
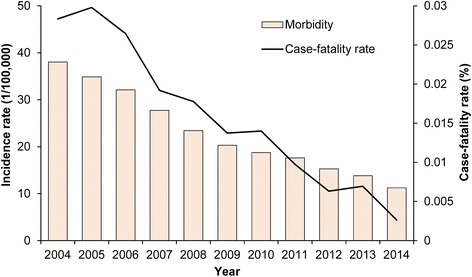
Incidence rate and case-fatality rate of bacillary dysentery in China, 2004–2014

The reported annual incidence and case-fatality rate of bacillary dysentery from 2004 to 2014 according to the NIDRS are shown in Fig. 1. During 2004–2014, 3,342,847 cases of bacillary dysentery were reported in China with an average incidence rate of 22.89 cases per 100,000 person-years. The annual incidence rate of bacillary dysentery decreased by approximately 3-fold from 38.03 cases per 100,000 person-years in 2004 to 11.24 cases per 100,000 person-years in 2014 (*Z* = −672.51, *P* < 0.05, by Cochran-Armitage trend test). During 2004–2014, a total of 636 deaths due to bacillary dysentery were reported, and the case-fatality rate of bacillary dysentery decreased from 0.028% in 2004 to 0.0026% in 2014.

### Demographic features

**Fig. 2:**
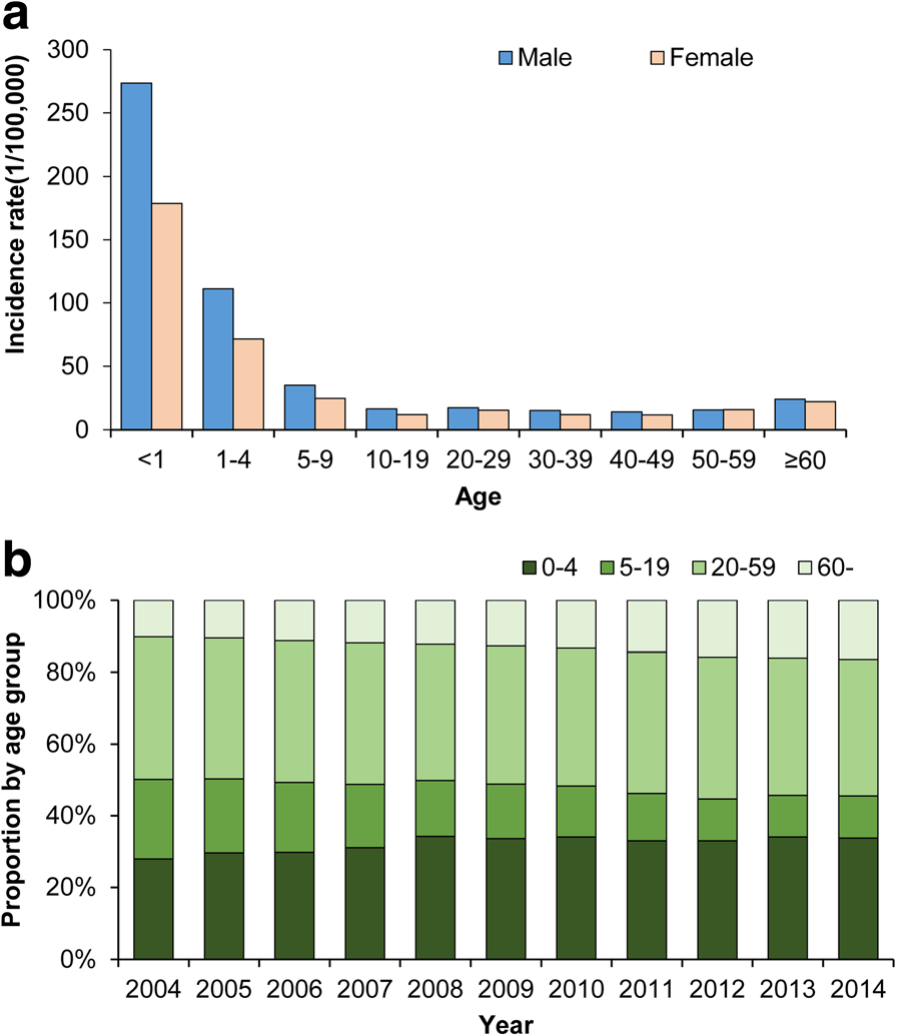
Age and sex distribution of bacillary dysentery in China, 2004–2014. **a** The average annual incidence of bacillary dysentery by age and sex was calculated by dividing the total number of cases occurring in a specific age group during 2004–2014 by the corresponding population and multiplying by 100,000. **b** The proportion of bacillary dysentery cases by age groups

The incidence rate of bacillary dysentery varied greatly according to age groups, with the highest rate being 228.59 cases per 100,000 person-years observed in children <1 years of age, followed by an average incidence rate of 92.58 cases per 100,000 person-years in children 1–4 years of age. Cases identified in children <5 years of age accounted for 31.47% of the total number of cases reported from 2004 to 2014. The data showed a male predominance in most age groups, with an overall male-to-female ratio of 1.31:1. Male predominance was most obvious within children <5 years of age, and the incidence of bacillary dysentery was 1.54 times higher in boys younger than 5 years than in girls of the same age (146.89 *vs* 93.46 cases per 100,000 person-years, *χ* ^*2*^ = 46272.89, *P* <0.05) (Fig. 2a). The proportion of bacillary dysentery cases that were identified in individuals <5 years of age or ≥60 years of age increased from 2004 to 2014 (<5 years of age: 27.85–33.77%, Cochran-Armitage trend test: *Z* = 77.76, *P* < 0.05; ≥60 years of age: 10.20–16.43%, Cochran-Armitage trend test: *Z* = 108.87, *P* < 0.05). In contrast, the proportion of cases identified in individuals 5–19 years of age decreased from 22.27% in 2004 to 11.73% in 2014 (*Z* = −175.18, *P* < 0.05, by Cochran-Armitage trend test) (Fig. 2b).

### Seasonal distribution

**Fig. 3:**
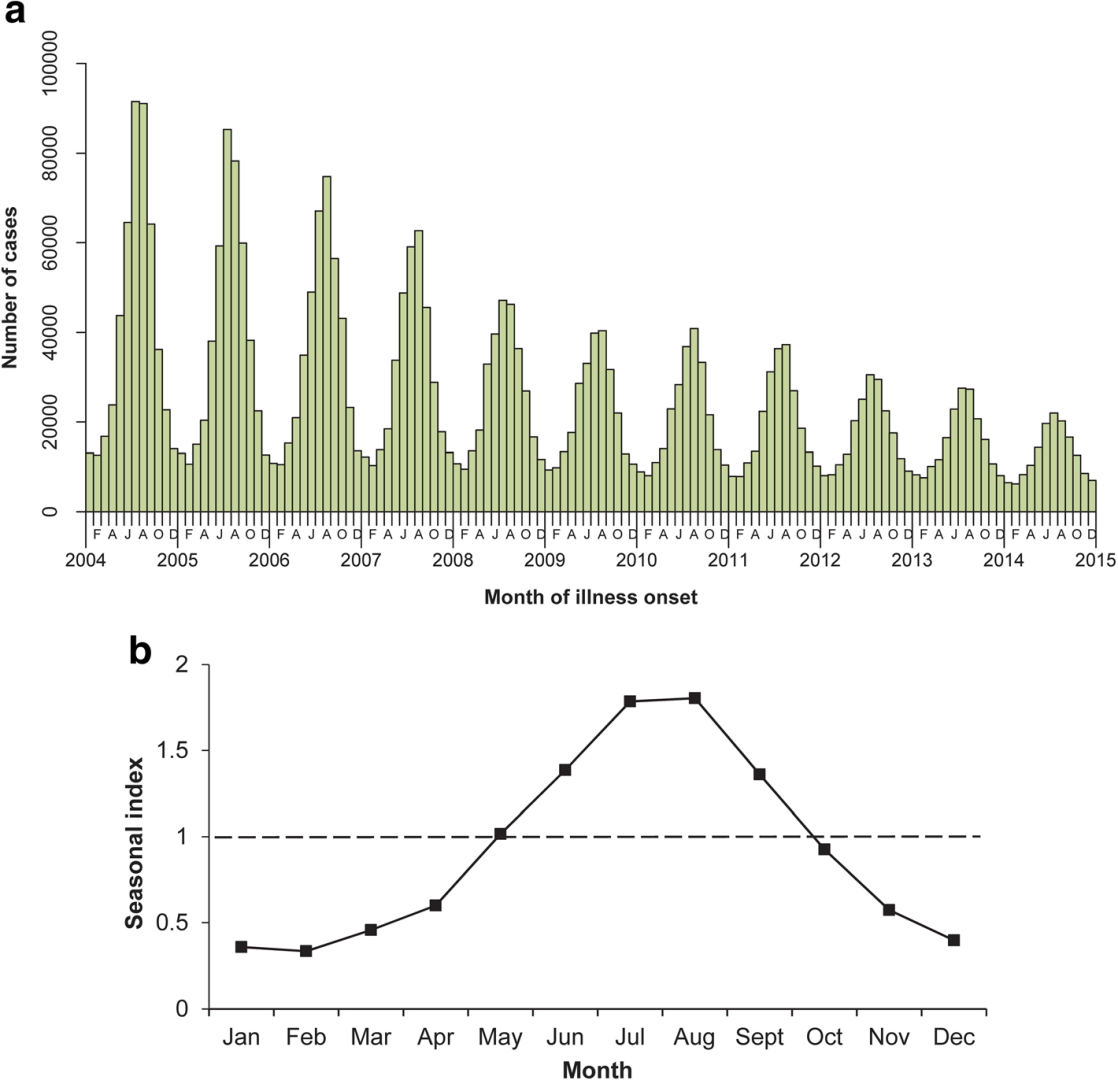
Seasonal distribution of bacillary dysentery cases in China, 2004–2014. **a** The frequency distribution of bacillary dysentery cases by month of illness onset. **b** The seasonal index of bacillary dysentery. The seasonal index was calculated by month as the average case count for a given month divided by the average monthly case count during the entire 11-year time period, 2004–2014. No obvious seasonal pattern was expected if the seasonal index of each month was close to 1.0

In China during 2004–2014, bacillary dysentery presented obvious seasonal characteristic (Fig. 3a, b). The majority of bacillary dysentery cases occurred from June to September, during which 57.60% of cases were reported. The seasonal index was highest in August (1.80) when the average monthly incidence rate was 41.97 per 100,000 person-years.

### Geographical distribution

**Fig. 4:**
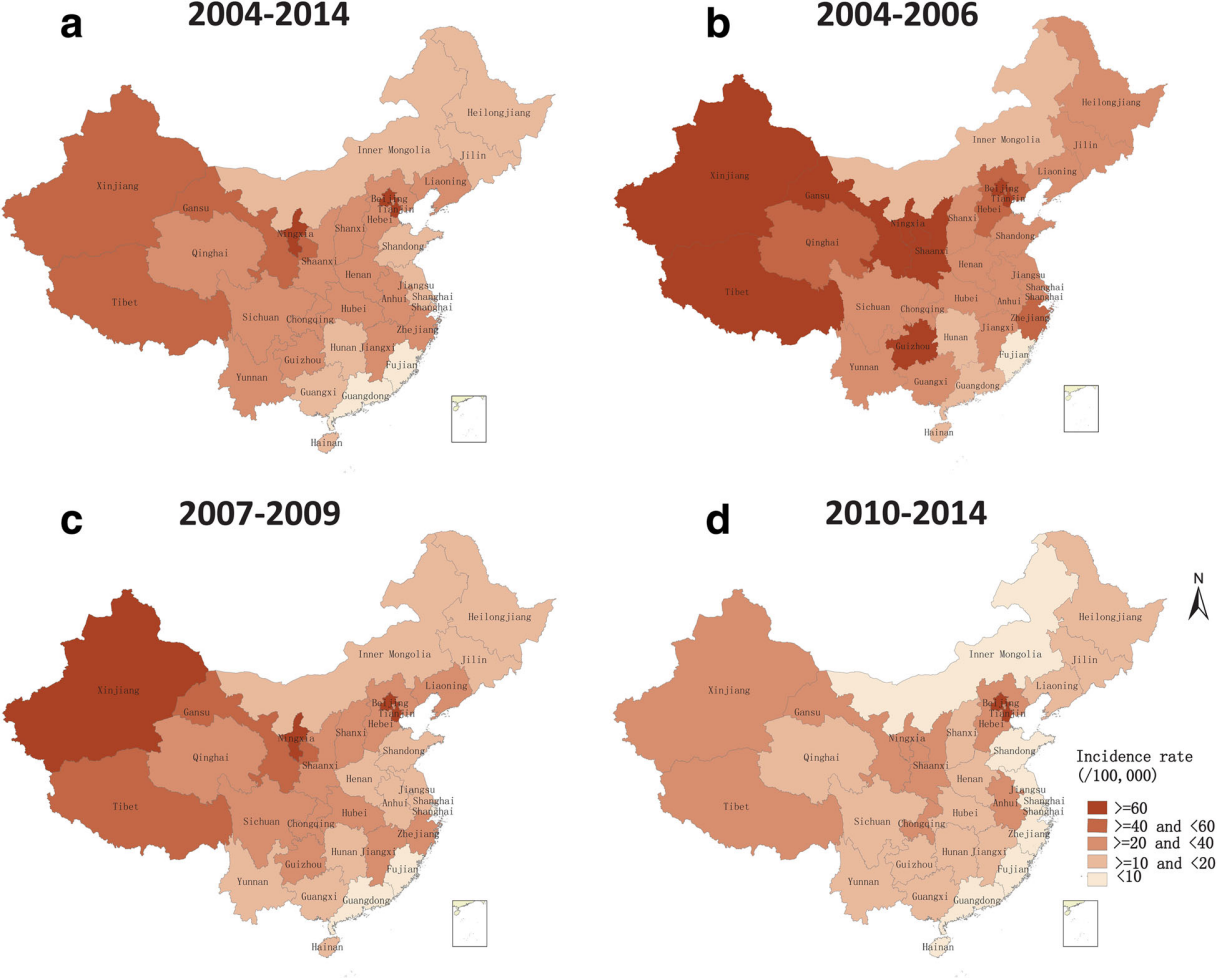
Spatial patterns of bacillary dysentery morbidity in China, 2004–2014. **a** Average incidence by province, 2004–2014. **b**–**d** Average incidence by province for three time periods (2004–2006 [**b**], 2007–2009 [**c**], and 2010–2014 [**d**]). Average incidence by province for different time periods was calculated by dividing the total number of cases in each time period by the corresponding population and multiplying by 100,000

Despite an overall decrease in incidence rate over the time period of the study, northwest China (Tibet, Xinjiang, Gansu, and Ningxia), Beijing, and Tianjin consistently had the highest incidence rates of bacillary dysentery from 2004 to 2014. Southeast China (Jiangsu, Shanghai, Fujian, Guangdong, and Hainan), northeast China (Jilin, Heilongjiang), and Inner Mongolia had relatively lower incidence rates of bacillary dysentery. The number of provinces with an incidence rate >60 per 100,000 person-years reduced from eight (Beijing, Ningxia, Tianjin, Tibet, Gansu, Xinjiang, Shannxi, and Guizhou) in 2004–2006 to two (Beijing and Tianjin) in 2010–2014. The number of provinces with an incidence rate <20 per 100,000 person-years progressively increased from five (Hainan, Inner Mongolia, Hunan, Guangdong, and Fujian) in 2004–2006 to 14 in 2007–2009 and to 21 in 2010–2014 (Fig. 4).

### Distribution of *Shigella* species that cause bacillary dysentery

**Fig. 5:**
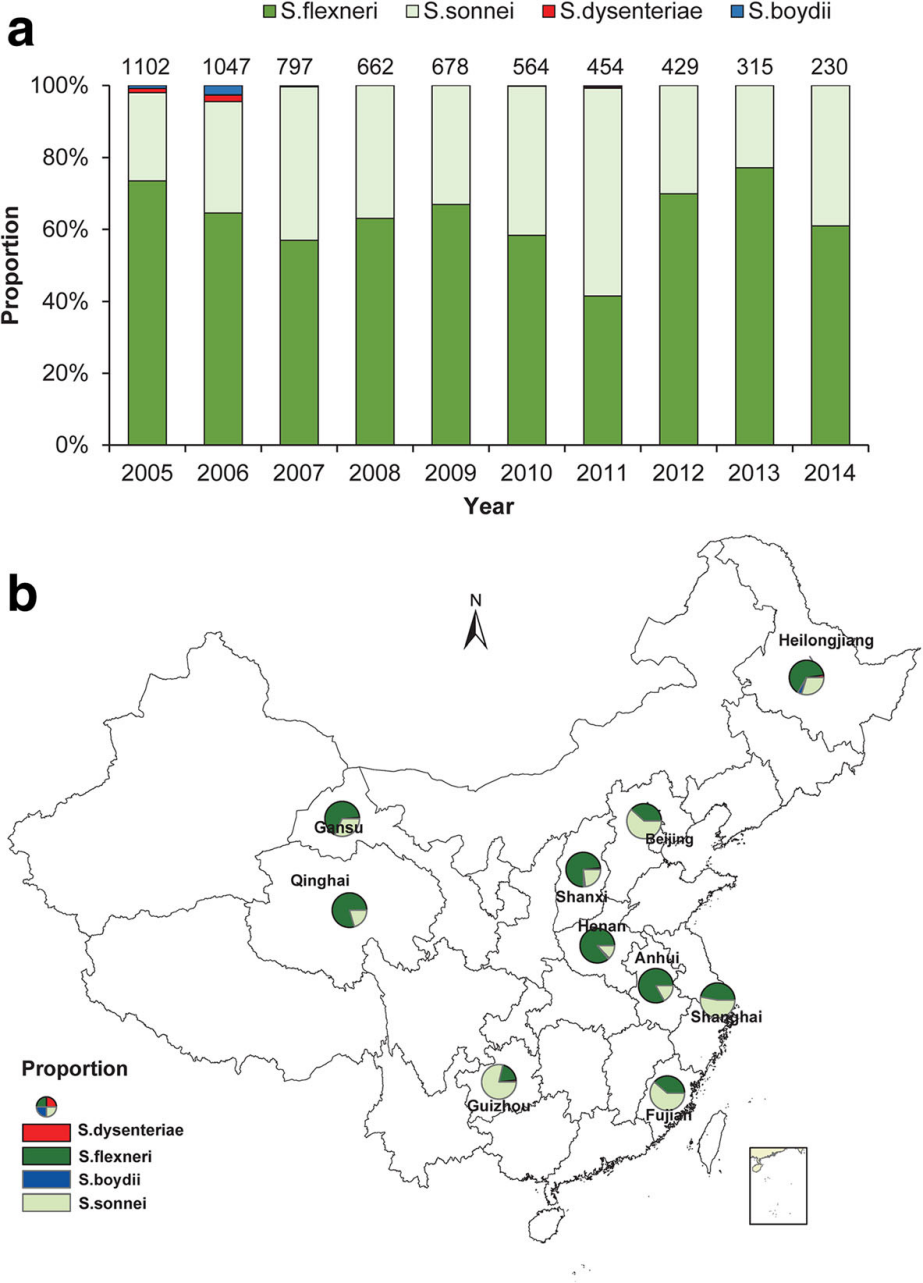
Proportion of *Shigella* spp. isolated in laboratory-confirmed cases of bacillary dysentery by year and province in sentinel hospitals, 2005–2014. **a** The temporal distribution of the proportion by *Shigella* spp. **b** The geographical distribution of the proportion by *Shigella* spp.

From 2005 to 2014, a total of 70,802 samples were collected and tested for the presence of *Shigella* bacteria in sentinel hospitals. From all tested samples, a total of 6278 *Shigella* isolates (8.87%) were isolated. Of these, *S. flexneri* was the most prevalent (4009 isolates, 63.86%), followed by *S. sonnei* (2191 isolates, 34.89%). *Shigella dysenteriae* (38 isolates) and *S. boydii* (40 isolates) were relatively uncommon (only present in 2005, 2006, 2007 and 2011) and accounted for only 0.61 and 0.63% of the isolates, respectively (Fig. 5a).

Figure 5b shows the geographical distribution of *Shigella* species determined by serotyping isolates obtained from patients with diarrhea and clinically suspected dysentery during 2005–2014. *Shigella flexneri* was the most prevalent in central and northern China except for in Beijing; *S. sonnei* was the most prevalent in eastern and southern China (Shanghai, Guizhou and Fujian).

### Antimicrobial resistance of *Shigella* isolates

**Fig. 6:**
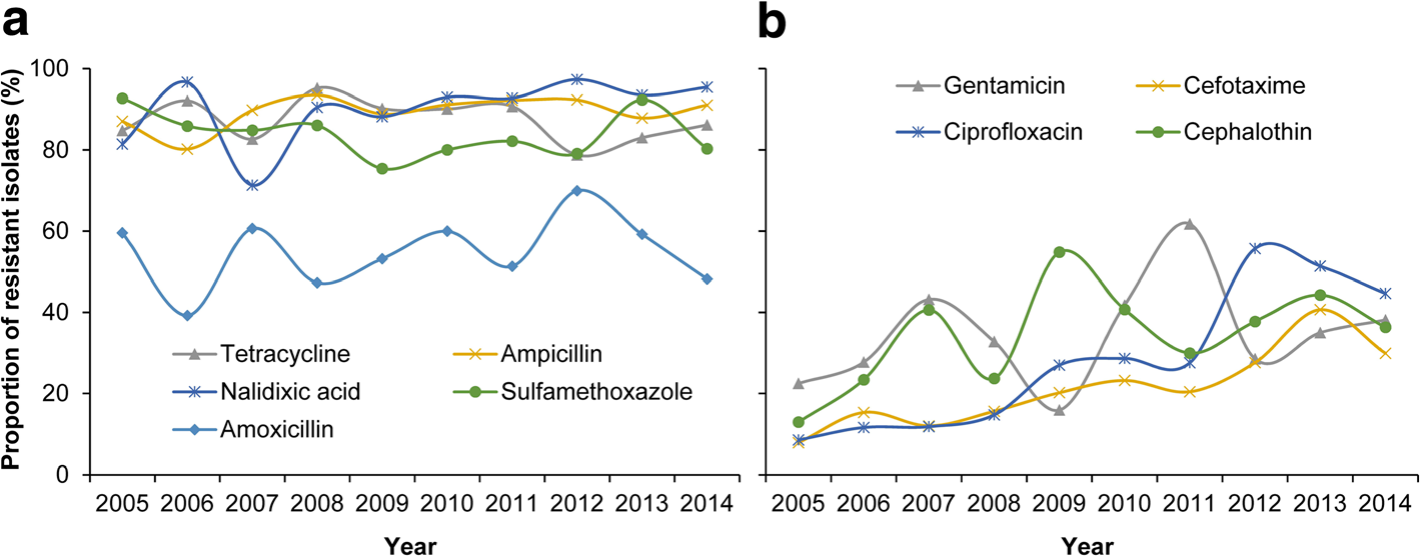
Proportion of *Shigella* isolates resistant to different antimicrobial drugs in China, 2005–2014. **a** Proportion of *Shigella* isolates resistant to nalidixic acid, ampicillin, tetracycline, sulfamethoxazole, and amoxicillin.** b** Proportion of *Shigella* isolates resistant to Cephalothin, Gentamicin, Ciprofloxacin, and Cefotaxime﻿. The proportion was calculated by dividing the number of isolates resistant to each antibiotic by all tested isolates

The resistance rates of *Shigella* during the study period are shown in Table 1. Isolates had highest resistance against nalidixic acid (89.13% of tested isolates resistant), followed by ampicillin (88.90%), tetracycline (88.43%), and sulfamethoxazole (82.92%). The proportion of isolates resistant to nalidixic acid, ampicillin, tetracycline, or sulfamethoxazole was consistently high during 2005–2014 (Fig. 6a﻿). Furthermore, the percentage of isolates resistant to ciprofloxacin and cefotaxime increased from 8.53 and 7.87% in 2005 to 44.65 and 29.94% in 2014 (*Z* = 18.31 and 10.22, *P* < 0.01, by Cochran-Armitage trend test), respectively (Fig. 6b).

**Table 1:** Antimicrobial resistance of *Shigella* isolates obtained in sentinel hospitals in China, 2005–2014

Antibiotic	Number of isolates tested	Percentage of resistant isolates (%)
Nalidixic acid	4178	89.13
Ampicillin	4335	88.90
Tetracycline	4247	88.43
Sulfamethoxazole	4223	82.92
Amoxicillin	4060	53.17
Cephalothin	3952	34.82
Gentamicin	4254	34.28
Ciprofloxacin	3583	22.10
Cefotaxime	4247	19.10

## Discussion

This study explored the morbidity, mortality, sex-specific annual incidence, and geographic and temporal distribution of bacillary dysentery in China using the surveillance dataset from the NIDRS during 2004–2014. In addition, the dominant *Shigella* species that cause bacillary dysentery along with their resistance to common antimicrobial drugs were analyzed using samples collected at sentinel hospitals during 2005–2014.

The incidence rate of bacillary dysentery during 2004–2014 in China showed an overall decline. The lowest rate (11.24 per 100,000 person-years) was observed in 2014, which was less than one third of the incidence rate in 2004 (38.03 per 100,000 person-years). A number of factors may have contributed to the observed decline, including rapid economic development, improvement of water supply systems, improved sanitary facilities, and a rising awareness of the importance of sanitation [16]. However, the number of cases as well as the incidence rate of bacillary dysentery in China remains higher than that in many developed countries, such as the USA, Australia, England and France, where the incidence of bacillary dysentery reported was 1.8–6.5 cases per 100,000 person-years [7]. Some regions in northwest China (including Tibet, Xinjiang, Gansu, Qinghai, and Ningxia) and in northern China (including Beijing and Tianjin) consistently showed higher morbidity than other regions in China during the study period. The high incidence rates of bacillary dysentery in Tibet, Xinjiang, Qinghai, Gansu, and Ningxia are consistent with their relatively low Gross Domestic Product [14]. These results indicate that economic development, which is tightly associated with the condition and accessibility of health facilities and healthcare resources, is potentially one of the major factors determining the occurrence of bacillary dysentery [17]. The high incidence rate observed in Beijing and Tianjin could potentially be explained by the high population density and good case ascertainment rates in these two regions [18]. These two regions are also known for having a large transient population who mostly live in suburban areas without proper sanitation and access to clean water [19].

In this study, bacillary dysentery was observed in all age groups, but individuals <5 years of age and individuals ≥60 years of age had higher incidence rates of bacillary dysentery in China. Unlike in other countries, the highest incidence rate was observed in infants <1 year of age [7, 20–23]. The finding suggests that additional studies are needed to statistically or mechanistically characterize risk factors of bacillary dysentery associated with breastfeeding infants in China. Children <5 years of age are likely susceptible to bacillary dysentery because of low immune function, lack of previous exposure, and a higher chance of exposure to a *Shigella*-contaminated environment through play-related activities [24, 25]. The difference in incidence between male and female individuals, especially those <5 years of age may be because male individuals are generally more active than female individuals, and thus have more opportunities to be exposed to environments containing bacteria [19]. Over the study period, we also observed a decrease in the proportion of bacillary dysentery cases identified in individuals 5–19 years of age and a corresponding increase in the proportion of cases in individuals ≥60 years of age. Such a shift in relative age-specific risk may be because the majority of individuals 5–19 years of age are school students. In the past decades, schools have strengthened preventive measures against bacillary dysentery by providing safe drinking water, safe food, and health education. All together, our findings suggest that preventive interventions to control bacillary dysentery should emphasize protection for individuals <5 years of age and individuals ≥60 years of age. Within each year, incidence rates of bacillary dysentery generally peaked between June and September. Previous studies have demonstrated that temperature plays an important role in the seasonal fluctuation of the incidence of bacillary dysentery as variation in temperature can impact the survival and reproduction of *Shigella* bacteria [26–31].

The distribution of species and serotypes of *Shigella* is dynamic over time and place. Based on our findings, the species distribution of *Shigella* in China was similar to that of other developing countries such as Bangladesh, Peru, and India [32–34]. Nonetheless, the distribution of *Shigella* species varied among different regions in China. Similar to developed countries, *S. sonnei* was the dominant species observed in Beijing, Shanghai, Guizhou, and Fujian. Beijing and Shanghai have undergone rapid economic development in recent years and represent the most developed regions in China. Interestingly, the dominant *Shigella* species in Beijing before 2006 was *S. flexneri* [35]. The transition of the predominant *Shigella* species from *S. flexneri* to *S.sonnei* was also observed in other fast-developing countries such as Thailand, Brazil, and Turkey, which suggests that regional socioeconomic development might predict the distribution of *Shigella* species. However, Guizhou and Fujian are located in southern China and have different meteorological conditions with high relative humidity and temperature [36].

Effective antimicrobial therapy of bacillary dysentery can significantly relieve symptoms and shorten both the duration of infection and the excretion time of bacteria [37]. In recent years, antimicrobial resistant *Shigella* strains have been commonly identified, threatening and complicating effective treatment of bacillary dysentery. Our study shows that *Shigella* isolated in China tend to have a higher resistance to nalidixic acid, ampicillin, tetracycline, and sulfamethoxazole than that found in many other countries [38, 39]. These antibiotics were previously used as first-line treatments and are still commonly prescribed to treat bacillary dysentery. From 2005 to 2014, there was also a gradual increase in the prevalence of *Shigella* bacteria resistant to cefotaxime and ciprofloxacin, which have become the first-line agents for treating bacillary dysentery in adults. Such increase in ciprofloxacin-resistant *Shigella* bacteria have also been observed in the United States [40, 41]. If introduced to populations in nursing homes or childcare settings, *Shigella* bacteria may spread rapidly and cause large protracted outbreaks. The fact that patterns of antimicrobial resistance of *Shigella* bacteria are continuously changing underscores the importance of continuous monitoring of antimicrobial susceptibility of *Shigella* bacteria and emphasizes how antimicrobial treatment of bacillary dysentery should be carried out according to antimicrobial susceptibility data.

This study has several limitations. First, most cases of bacillary dysentery with mild symptoms can be cured by oral antibiotics. Therefore, under-reporting may have occurred. Second, differences in health care seeking behaviours and access to health care may exist across different provinces, which may result in information bias. Third, this study is an analysis on routine surveillance data, and we were unable to collect detailed data of different *Shigella* species to examine the differences between them in terms of epidemic characteristics, clinical symptoms, and antimicrobial resistance.

## Conclusions

Bacillary dysentery has a considerable and continuing disease burden in China. However, the incidence rate of bacillary dysentery has been decreasing, presumably due to socioeconomic development, implementation of sanitation facilities, and health education. Children <5 years of age, particularly infants <1 year of age, have a higher incidence rate of bacillary dysentery than individuals in other age groups. Different regions in China differ in their peak incidence time. The incidence rate of bacillary dysentery remains high in northwest China. Corresponding to an extensive use of antimicrobial drugs, the proportion of antimicrobial-resistant *Shigella* bacteria is increasing, threatening effective treatment of bacillary dysentery. Priority interventions should be delivered to populations in northwest China and individuals <5 years of age. Effective treatment of bacillary dysentery must be guided by continuous monitoring of antibiotic resistance patterns.

## Abbreviations

ATCC: American Type Culture Collection
CDC: Centers for Disease Control and Prevention
CLSI: Clinical Laboratory Standards Institute
HPF: High power field
NIDRS: National Infectious Disease Information Reporting System

## References

[1] Nandy S, Dutta S, Ghosh S, Ganai A, Rajahamsan J, Theodore RB, Sheikh NK (2011). Foodborne-associated *Shigella sonnei*, India, 2009 and 2010. Emerg Infect Dis.

[2] Nygren BL, Schilling KA, Blanton EM, Silk BJ, Cole DJ, Mintz ED (2013). Foodborne outbreaks of shigellosis in the USA, 1998–2008. Epidemiol Infect.

[3] Bhattacharya SK, Sur D, Mahalanabis D (2012). Public health significance of shigellosis. Indian Pediatr.

[4] Centers for Disease Control and Prevention (CDC) (2013). Notes from the field: Outbreak of infections caused by *Shigella sonnei* with decreased susceptibility to azithromycin—Los Angeles, California, 2012. MMWR Morb Mortal Wkly Rep.

[5] He F, Han K, Liu L, Sun W, Zhang L, Zhu B, Ma H (2012). Shigellosis outbreak associated with contaminated well water in a rural elementary school: Sichuan Province, China, June 7–16, 2009. PLoS One.

[6] Niyogi SK (2005). Shigellosis. J Microbiol.

[7] Kotloff KL, Winickoff JP, Ivanoff B, Clemens JD, Swerdlow DL, Sansonetti PJ, Adak GK, Levine MM (1999). Global burden of *Shigella* infections: implications for vaccine development and implementation of control strategies. Bull World Health Organ.

[8] Pelczar MJ, Chan ECS (1981). Elements of microbiology.

[9] Ashkenazi S, Levy I, Kazaronovski V, Samra Z (2003). Growing antimicrobial resistance of *Shigella* isolates. J Antimicrob Chemother.

[10] Yang W, Li Z, Lan Y, Wang J, Ma J, Jin L, Sun Q, Lv W, Lai S, Liao Y (2011). A nationwide web-based automated system for outbreak early detection and rapid response in China. West Pac Surveill Response J.

[11] Ministry of Health of the People’s Republic of China (2008). Diagnostic Criteria for Bacillary and Amoebic Dysentery (WS 287-2008).

[12] National Health and Family Planning Commission. National Surveillance Project of Shigellosis. 2005. (in Chinese). http://www.nhfpc.gov.cn/zhuzhan/zcjd/201304/ac06031652644d308429d8a5a36ff443.shtml. Assessed 9 Apr 2015.

[13] Clinical and Laboratory Standards Institute. Performance standards for antimicrobial susceptibility testing. approved standard, 14th edn, document M100-S14. 2004.

[14] National Bureau of Statistics of China. National census in China. (in Chinese). http://data.stats.gov.cn/easyquery.htm?cn = E0103. Assessed 9 Apr 2015.

[15] Xiao D, Long Y, Wang S, Wu K, Xu D, Li H, Wang G, Yan Y (2012). Epidemic distribution and variation of Plasmodium falciparum and Plasmodium vivax malaria in Hainan, China during 1995–2008. Am J Trop Med Hyg.

[16] Wang XY, Du L, Von Seidlein L, Xu ZY, Zhang YL, Hao ZY, Han OP, Ma JC, Lee HJ, Ali M (2005). Occurrence of shigellosis in the young and elderly in rural China: results of a 12-month population-based surveillance study. Am J Trop Med Hyg.

[17] Ferrer SR, Strina A, Jesus SR, Ribeiro HC, Cairncross S, Rodrigues LC, Barreto ML (2008). A hierarchical model for studying risk factors for childhood diarrhoea: a case-control study in a middle-income country. Int J Epidemiol.

[18] Gao TLG, Li X, Jia L, Liu Y (2007). Analysis about epidemic situation of dysentery near upon fourteen years in Beijing. Chin J Preve Med.

[19] Xiao G, Xu C, Wang J, Yang D, Wang L (2014). Spatial-temporal pattern and risk factor analysis of bacillary dysentery in the Beijing-Tianjin-Tangshan urban region of China. BMC Public Health.

[20] Finkelstein Y, Moran O, Avitzur Y, Nussinovitch M, Harel L, Volovitz B, Amir J (2002). Clinical dysentery in hospitalized children. Infection.

[21] Edwards BH (1999). Salmonella and *Shigella* species. Clin Lab Med.

[22] Prado V, Lagos R, Nataro JP, San Martin O, Arellano C, Wang JY, Borczyk AA, Levine MM (1999). Population-based study of the incidence of *Shigella* diarrhea and causative serotypes in Santiago, Chile. Pediatr Infect Dis J.

[23] Yurdakok K, Sahin N, Ozmert E, Berkman E (1997). *Shigella* gastroenteritis: clinical and epidemiological aspects, and antibiotic susceptibility. Acta Paediatr Jpn.

[24] Ahmed K, Shakoori FR, Shakoori AR (2003). Aetiology of shigellosis in northern Pakistan. J Health Popul Nutr.

[25] Hossain MA, Albert MJ, Hasan KZ (1990). Epidemiology of shigellosis in Teknaf, a coastal area of Bangladesh: a 10-year survey. Epidemiol Infect.

[26] Gao L, Zhang Y, Ding G, Liu Q, Zhou M, Li X, Jiang B (2014). Meteorological variables and bacillary dysentery cases in Changsha City, China. Am J Trop Med Hyg.

[27] Ma W, Sun X, Song Y, Tao F, Feng W, He Y, Zhao N, Yuan Z (2013). Applied mixed generalized additive model to assess the effect of temperature on the incidence of bacillary dysentery and its forecast. PLoS One.

[28] Zhang Y, Bi P, Hiller JE, Sun Y, Ryan P (2007). Climate variations and bacillary dysentery in northern and southern cities of China. J Infect.

[29] Checkley W, Epstein LD, Gilman RH, Figueroa D, Cama RI, Patz JA, Black RE (2000). Effect of El Nino and ambient temperature on hospital admissions for diarrhoeal diseases in Peruvian children. Lancet.

[30] Guan P, Huang D, Guo J, Wang P, Zhou B (2008). Bacillary dysentery and meteorological factors in northeastern China: a historical review based on classification and regression trees. Jpn J Infect Dis.

[31] Huang D, Guan P, Guo J, Wang P, Zhou B (2008). Investigating the effects of climate variations on bacillary dysentery incidence in northeast China using ridge regression and hierarchical cluster analysis. BMC Infect Dis.

[32] Ram PK, Crump JA, Gupta SK, Miller MA, Mintz ED (2008). Part II. Analysis of data gaps pertaining to *Shigella* infections in low and medium human development index countries, 1984–2005. Epidemiol Infect.

[33] Fernandez-Prada CM, Venkatesan MM, Franco AA, Lanata CF, Sack RB, Hartman AB, Spira W (2004). Molecular epidemiology of *Shigella flexneri* in a diarrhoea-endemic area of Lima, Peru. Epidemiol Infect.

[34] Pazhani GP, Ramamurthy T, Mitra U, Bhattacharya SK, Niyogi SK (2005). Species diversity and antimicrobial resistance of *Shigella* spp. isolated between 2001 and 2004 from hospitalized children with diarrhoea in Kolkata (Calcutta), India. Epidemiol Infect.

[35] Mao Y, Cui E, Bao C, Liu Z, Chen S, Zhang J, Wang H, Zhang C, Zou J, Klena JD (2013). Changing trends and serotype distribution of *Shigella* species in Beijing from 1994 to 2010. Gut Pathog.

[36] Chompook P, Samosornsuk S, von Seidlein L, Jitsanguansuk S, Sirima N, Sudjai S, Mangjit P, Kim DR, Wheeler JG, Todd J (2005). Estimating the burden of shigellosis in Thailand: 36-month population-based surveillance study. Bull World Health Organ.

[37] Sur D, Ramamurthy T, Deen J, Bhattacharya SK (2004). Shigellosis : challenges & management issues. Indian J Med Res.

[38] Ozmert EN, Ince OT, Orun E, Yalcin S, Yurdakok K, Gur D (2011). Clinical characteristics and antibiotic resistance of *Shigella* gastroenteritis in Ankara, Turkey between 2003 and 2009, and comparison with previous reports. Int J Infect Dis.

[39] Pourakbari B, Mamishi S, Mashoori N, Mahboobi N, Ashtiani MH, Afsharpaiman S, Abedini M (2010). Frequency and antimicrobial susceptibility of *Shigella* species isolated in Children Medical Center Hospital, Tehran, Iran, 2001–2006. Braz J Infect Dis.

[40] Bowen A, Hurd J, Hoover C, Khachadourian Y, Traphagen E, Harvey E, Libby T, Ehlers S, Ongpin M, Norton JC (2015). Importation and domestic transmission of *Shigella sonnei* resistant to ciprofloxacin-United States, May 2014–February 2015. MMWR Morb Mortal Wkly Rep.

[41] Folster JP, Pecic G, Bowen A, Rickert R, Carattoli A, Whichard JM (2011). Decreased susceptibility to ciprofloxacin among *Shigella* isolates in the United States, 2006 to 2009. Antimicrob Agents Chemother.

